# Indentification of single base mutations in the GCK gene of patients with diagnosis of MODY2

**DOI:** 10.1186/1758-5996-7-S1-A220

**Published:** 2015-11-11

**Authors:** Francisco Geraldo Mello da Rocha Carvalho Neto, Danielle Rachel dos Santos Carvalho, Simone Bruggemann Mota, Adolfo José da Mota, Deborah Laredo Jezini, Tetsuo Yamane

**Affiliations:** 1 Universidade Federal do Amazonas – UFAM, Manaus, Brazil

## Background

Diabetes mellitus (DM) refers to a group of common metabolic disorders, with multiple causes, that share a common phenotype: hyperglycemia (Kota, 2012). The concept of multiple etiologies is recent and a third group of DM is proposed today, a monogenic form with autosomal dominant inheritance: MODY. There are 13 subtypes described for MODY, which in MODY2 is one of the most common, although the relative frequency varies according to the study population (Corrales et al., 2010). MODY2 is provoked by mutations in the glucokinase gene (GCK) located on human chromosome 7p15.3-p15.1, which consists of 10 exons that span 45.169 bp and encode a 465-amino-acid protein (Tinto et al., 2008).

## Objective

To identify single base mutations in the GCK gene of patients with diagnosis of MODY2 and evaluate whether these mutations are related to the disease.

## Materials and methods

This study was approved by Ethics Committee under no 923744. Patients who provided blood samples signed informed consent. DNA was extracted with Genomic DNA mini kit (Invitrogen). Primers for amplification of exon 10 are described in the literature (Boutin et al., 2001) and PCR was performed with GoTaq kit (Promega). The amplicons were sequenced with BigDye Terminator v.3.1 kit (Life Technologies). Sequences obtained were analyzed by SeqManTM II program (DNA Star Inc.). Comparisons of the files were held with the bank sequences of the human genome plus the bank transcripts (NCBI) using BLAST program (Altschul et al, 1997).

## Results

The case study was conducted with a female proband, 40 yrs., with MODY2 diagnosis. Santos et al (2014) identified 3 mutations in the upstream promoter GCK gene for this proband and hypothesized if mutations in the promoter could be responsible for the disease. To test this hypothesis, we started sequencing all exons to exclude the possibility if other mutations are responsible for the phenotype. So far, we haven't found SNPs in sequenced exons (A, B and C) and from 2 to 6. Few studies have reported mutations in GCK promoter, but these can be connected with some form of diabetes, such as gestational diabetes (Santos et al 2014).

## Conclusions

In this study, we sequenced 70% of the exons of the GCK gene and SNPs were not identified or any other type of mutation that corroborate the hypothesis of MODY2. However, these Results added to the previous GCK promoter data of this proband strengthens the hypothesis that the reported mutations interfere with the ideal regulation of gene expression.

